# Analysis of acute vascular damage after photodynamic therapy using benzoporphyrin derivative (BPD)

**DOI:** 10.1038/sj.bjc.6690271

**Published:** 1999-04

**Authors:** V H Fingar, P K Kik, P S Haydon, P B Cerrito, M Tseng, E Abang, T J Wieman

**Affiliations:** Department of Surgery, Division of Surgical Oncology, Department of Mathematics, Department of Anatomy and Neurobiology, University of Louisville, Louisville, KY, 40292, USA

**Keywords:** photodynamic therapy, BPD, vascular effects, chondrosarcoma

## Abstract

Benzoporphyrin derivative monoacid ring A (BPD-MA, verteporfin) is currently under investigation as a photosensitizer for photodynamic therapy (PDT). Since BPD exhibits rapid pharmacokinetics in plasma and tissues, we assessed damage to tumour and muscle microvasculature when light treatment for PDT was given at short times after injection of photosensitizer. Groups of rats with chondrosarcoma were given 2 mg kg^−1^ of BPD intravenously 5 min to 180 min before light treatment of 150 J cm^−2^ 690 nm. Vascular response was monitored using intravital microscopy and tumour cure was monitored by following regrowth over 42 days. For treatment at 5 or 30 min after BPD injection, blood flow stasis was limited to tumour microvasculature with lesser response in the surrounding normal microvasculature, indicating selective targeting for damage. No acute changes were observed in vessels when light was given 180 min after BPD injection. Tumour regression after light treatment occurred in all animals given PDT with BPD. Long-term tumour regression was greater in animals treated 5 min after BPD injection and least in animals given treatment 180 min after drug injection. The correlation between the timing for vascular damage and cure implies that blood flow stasis plays a significant role in PDT-induced tumour destruction. © 1999 Cancer Research Campaign


*British Journal of Cancer* (1999) **79**(11/12), 1702–1708
© 1999 Cancer Research Campaign
Article no. bjoc.1998.0271

### Analysis of acute vascular damage after photodynamic therapy using benzoporphyrin derivative (BPD)

**VH Fingar[1], PK Kik[1], PS Haydon[1], PB Cerrito[2], M Tseng[3], E Abang[3] and TJ Wieman[1]**

[1]Department of Surgery, Division of Surgical Oncology, [2]Department of Mathematics, [3]Department of Anatomy and Neurobiology, University of Louisville, Louisville, KY 40292, USA

**Summary** Benzoporphyrin derivative monoacid ring A (BPD-MA, verteporfin) is currently under investigation as a photosensitizer for photodynamic therapy (PDT). Since BPD exhibits rapid pharmacokinetics in plasma and tissues, we assessed damage to tumour and muscle microvasculature when light treatment for PDT was given at short times after injection of photosensitizer. Groups of rats with chondrosarcoma were given 2 mg $kg^{-1}$ of BPD intravenously 5 min to 180 min before light treatment of 150 J $cm^{-2}$ 690 nm. Vascular response was monitored using intravital microscopy and tumour cure was monitored by following regrowth over 42 days. For treatment at 5 or 30 min after BPD injection, blood flow stasis was limited to tumour microvasculature with lesser response in the surrounding normal microvasculature, indicating selective targeting for damage. No acute changes were observed in vessels when light was given 180 min after BPD injection. Tumour regression after light treatment occurred in all animals given PDT with BPD. Long-term tumour regression was greater in animals treated 5 min after BPD injection and least in animals given treatment 180 min after drug injection. The correlation between the timing for vascular damage and cure implies that blood flow stasis plays a significant role in PDT-induced tumour destruction.

**Keywords:** photodynamic therapy; BPD; vascular effects, chondrosarcoma

Photodynamic therapy (PDT) is a rapidly evolving treatment modality with uses ranging from oncology, cardiovascular disease to hyperproliferative disorders. PDT relies on the ability of certain photosensitizing agents, usually porphyrins, to absorb light and convert this energy into cytotoxic molecules including singlet oxygen. The therapeutic value of this treatment is defined by the location of photosensitizer during light treatment.

The initial photosensitizer to enter clinical trials, Photofrin, has been recently approved for use in the USA, Canada, and abroad for treatment of solid tumours and new drugs are in various stages of pre-clinical and clinical development. Benzoporphyrin derivative monoacid ring A (BPD-MA, verteporfin) is a second generation photosensitizer that has several favourable characteristics for use in PDT over Photofrin (Levy, 1994). This agent is chemically pure and is activated with light at long wavelengths (690 nm) which allows theoretically for deeper penetration of light into the treatment area. Benzoporphyrin derivative has rapid plasma and tissue pharmacokinetics and is thus quickly cleared from tissues, resulting in a diminished period of skin photosensitivity compared to other drugs including Photofrin (Richter et al, 1990; Levy et al, 1993). A wide range of diseases are under investigation for treatment with PDT using BPD including solid tumours (Richter et al, 1993), choroidal neovascularization (Lin et al, 1994), arthritis (Chowdary, 1994), HIV therapy (North et al, 1996) and uterine endometrial ablation (Wyss et al, 1994).

Although the targets for BPD based PDT-induced destruction of tissue remain largely unresolved, studies by Schmidt-Erfurth et al (1995) have demonstrated vascular damage leading to blood flow stasis. The predominance of vascular damage in treated tissues correlates well with the high serum levels of photosensitizer that are present at short times after drug injection. Light treatment is generally given within 3 h of drug injection because of the rapid pharmacokinetics of BPD in tissue (Richter et al, 1990). Tissue microvasculature is an important target for PDT using several photosensitizers (Henderson and Farrell, 1989; Fingar et al, 1992) and direct damage to cells in the vessel wall including endothelial cells, and damage to blood components, including platelets, has been observed (Henderson and Dougherty, 1992).

Experiments were performed to evaluate the relative effectiveness of PDT using BPD at relatively short and long times after injection. The time between drug injection and light dose was varied in attempt to elucidate any differences in treatment efficacy and tissue selectivity at short intervals when the drug is chiefly in circulation, versus longer time intervals, when the drug has time to partition into the cells. Traditionally, solid tumours have been treated 180 min after injection of 2 mg $kg^{-1}$ BPD (Allison et al, 1991; Richter et al, 1991); however, non-oncological treatments for PDT such as neovascular ablation have determined that shorter intervals between BPD injection and light treatment offered better vascular uptake of photosensitizer (Haimovici et al, 1997). A goal of these studies was to determine whether shorter intervals between injection and light treatment would increase tumour response.

*Received 19 June 1998*
*Revised 1 September 1998*
*Accepted 2 September 1998*

*Correspondence to:* VH Fingar

### MATERIALS AND METHODS

### Animal model

Male Sprague-Dawley rats (80–100 g, Harlan Sprague–Dawley Inc., Indianapolis, IN, USA) were obtained at least 1 week before experimentation and were housed in a temperature-controlled environment with controlled light/dark cycles. Animals were fed standard laboratory chow and water ad libitum. Approval for this project was obtained from the Animal Care and Use Committee at the University of Louisville and was in compliance with National Institute of Health guidelines.

A chondrosarcoma tumour cell line maintained in our laboratory was used in these experiments as described previously (Fingar et al, 1990). For tumour implantation in skin, 0.3 ml ($1 \times 10^6$ cells $ml^{-1}$) of tumour cells were injected subcutaneously in the centre of the right flank for treatment response experiments with a 23 gauge needle. Tumours were used when they reached a diameter of 6–7 mm which occurred within 3–5 days after implantation. A separate series of animals were given tumour in both the right and left flank for experiments of fluorescein dye exclusion after PDT.

For tumour implantation into cremaster muscle, rats (80–120 g) were anaesthetized with sodium pentobarbital (50 mg $kg^{-1}$ i.p.). The right testicle was then pushed into the abdominal cavity and a lower midline laparatomy was performed under sterile conditions and the cremaster was gently retracted. A chondrosarcoma suspension was prepared from donor animals and 0.1 ml ($1 \times 10^5$ cells) of this suspension was injected into the muscle with a 27 gauge needle. Tumours were used for experimentation when they had reached a surface diameter of 2–3 mm and a thickness of 1 mm (3–4 days after implantation).

### Photosensitizer

The animals were given BPD-MA in a liposomal formulation (obtained as a gift from QLT Phototherapeutics, Vancouver, Canada) via tail vein injection at a dose of 2 mg $kg^{-1}$. The liposomal BPD was supplied as a lyophilized powder and was initially suspended in distilled water to a stock concentration of 1.47 mg $ml^{-1}$. This solution was further diluted to 0.39 mg $ml^{-1}$ using a 5% dextrose solution and was used within 14 days of preparation. Drug concentrations were verified by absorbance spectroscopy.

### Light treatment

Rats were anaesthetized with 50 mg $kg^{-1}$ sodium pentobarbital i.p. and placed on their left side. The right leg was shaved and depilated, then fixed during light treatment to prevent leg movement. For photosensitizer activation, an Argon-dye laser (Laser Ionics model 1400-12A, or a Coherent Innova 200, Palo Alto, CA, USA) and a dye laser (Spectra-Physics model 375b dye laser, Mountain View, CA, USA) with a fibreoptic light delivery system and microlens was used to illuminate the treatment field containing tumour. The wavelength was adjusted to 690 nm and verified by scanning monochromator (Optometrics model DMC1-02, Aver, MA, USA). The power density of light was 75 mW $cm^{-2}$ measured by a thermopile (Coherent model 210, Auburn, CA, USA) for a total dose of 150 J $cm^{-2}$ light (2000 s treatment time).

### Histological analysis of vessel damage

Tissue samples of cremaster muscle from animals given light treatment at 5 min, 30 min and 180 min after BPD injection or in sham controls given light treatment alone were collected for histological study. Cremaster muscles were fixed in situ by flooding the tissue with a pre-warmed fixative of 4% buffered formalin containing 0.25% glutaraldehyde. The entire cremaster muscle was then removed and immersed in the same formalin–glutaraldehyde solution for 24 h at 4°C. Tissue segments containing pairs of arterioles and venules were identified and dissected free of surrounding muscle with the aid of a microscope. Samples were processed in 1% osmium tetroxide, dehydrated, embedded in araldite 502, polymerized and sectioned at 1 µm. Sections were stained with toluidine blue before examination.

### Cremaster muscle preparation for normal and tumour microvascular studies

Rats were anaesthetized with sodium pentobarbital (55 mg $kg^{-1}$, i.p.) and placed on their backs on a temperature-controlled heating pad. Rectal temperature was maintained at 37°C and back temperature was monitored with a thermocouple to avoid local overheating of the skin. The right cremaster muscle was prepared for microvascular observations in the manner previously reported (Fingar et al, 1992). The muscle was slit on the ventral midline and spread with sutures over a cover glass which was positioned on a cylindrical pedestal. The cremaster was flooded with saline and covered with a clear plastic film to prevent drying. Transmitted light video microscopy was used to visualize normal muscle and tumour microvasculature. Images were recorded on VHS videotape for analysis. Arteriole and venule pairs in normal muscle were chosen for the study based on their diameter (20–30 µm) and branching order (fourth order). Vessels in tumour were chosen on size alone since there was no distinct difference in morphology between 20 and 30 µm tumour arterioles and venules.

Four to five animals were used in each different experimental group for measurement of vessel diameters. Red blood cell column diameter (RBCC diameter) and vessel wall diameter were measured every 5 min for the 33 min of light treatment. Vessels were monitored for an additional 60 min at 10-min intervals following light therapy. The RBCC diameter is a measurement of the inner lumen of the vessel. Reductions in RBCC diameter without a change in wall diameter indicate that aggregates have formed at the inner vessel wall.

### Fluorescein exclusion study

Exclusion of fluorescein dye in tumour and surrounding skin was monitored in animals to determine if vessel stasis had occurred within 24 h after the completion of PDT treatment and was used to confirm the results from the cremaster studies. Animals given BPD were anaesthetized using 50 mg $kg^{-1}$ sodium pentobarbital and were given light treatment of 150 J $cm^{-2}$ to the tumour on the right flank as described above. The tumour on the left leg in this series of animals served as a control. Animals were injected i.v. with 0.3 ml of 10 mg $kg^{-1}$ sodium fluorescein solution in saline at either 0 h or 24 h after the completion of light treatment. Animals were sacrificed after 5 min. The location of fluorescein dye was monitored by exposing the tissue to ultraviolet light (Spectroline model MB-100 Westbury, NY, USA). Images in both PDT-treated and control tumours were recorded on photographic film.

### Gray scale analysis

A second set of animals were treated similarly with PDT to the above exclusion study and were given fluorescein dye either 0 h or 24 h after the completion of light treatment. Both the treated and control tumours were excised after 5 min and were sandwiched between two glass microscope slides using a 1 mm spacer to ensure a uniform thickness of tumour tissue. Fluorescence

© Cancer Research Campaign 1999

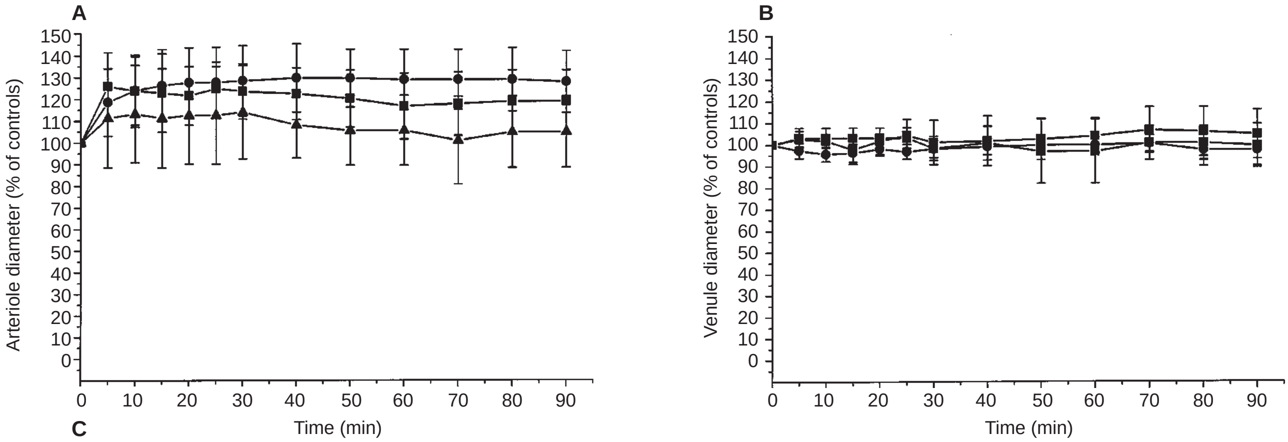

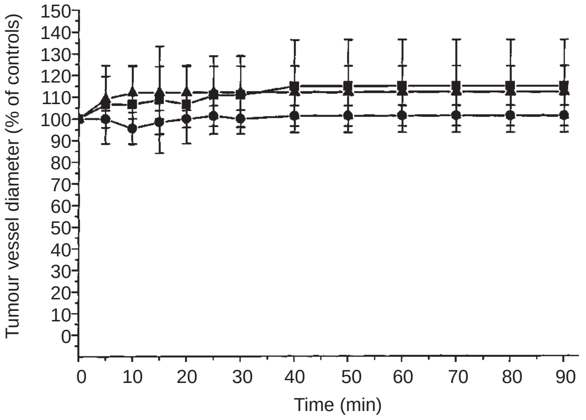

**Figure 1** Changes in vessel diameter following PDT with BPD. Animals were injected with 2 mg $kg^{-1}$ BPD: 5 min, 30 min, or 180 min before the initiation of light treatment. Light treatment was 150 J $cm^{-2}$ 690 nm to the cremaster muscle over the first 33 min of observation. The diameter of the outside wall of arterioles (**A**), venules (**B**), and tumour microvasculature (**C**) was measured at selected intervals during light treatment and for an additional 1 h after the completion of the light treatment. ( •) Five-minute time point, (■) 30-min time point data, (▲) 180-min time point data. Points represent the mean of 5–11 animals, measured as a percentage of the initial wall diameter. Bars represent 2 s.e.m.

microscopy images were recorded on VHS videotape from a Zeiss 20T microscope with a 4× objective and SIT camera (Hamamatsu model C2400–08, Bridgewater, NJ, USA). Epi-illumination using blue light (450-490 nm) was used to stimulate the fluorescein in the tissue for brief periods (< 5 s) and the resulting images were recorded. The camera settings were standardized between samples with a 10 ng $ml^{-1}$ fluorescein sample. Video images were digitized at a resolution of 512 × 512 pixels and the average gray level in 20 000 pixel area centred on tumour was calculated using a hand-written image analysis software package. Larger gray level data indicate greater levels of fluorescein fluorescence. Spatial uniformity of fluorescence intensity in fluorescein test samples was confirmed for the microscope sample field used for these studies before each experiment. Linearity between fluorescence intensity and gray level over the range of fluorescence values obtained in these studies was verified by imaging fluorescein samples ranging between 0.01 and 100 ng $ml^{-1}$ fluorescein. Three replicate images from PDT-treated animals and controls were taken for gray scale analysis.

### Tumour response monitoring

Animals were monitored for tumour regression and/or regrowth daily for 14 days and at weekly intervals afterwards for a total of 42 days after light treatment. Tumour cure was defined as animals with no detectable mass through 42 days. The effect of treatment on tumour and surrounding normal tissue was recorded.

### Statistics

The data were measured at each observation interval (13 points) during intravital microscopy for wall diameter and RBCC diameter in arterioles, venules and tumour microvasculature. The data were analysed using repeated measures analysis of variance to examine for differences in response at each time of treatment. Where the analysis of variance (ANOVA) showed statistical significance, least squares means were examined to compare the individual treatments pairwise. For measurements of fluorescence and gray scale analysis of tissue, the means from experiments in 3–4 animals and the corresponding 95% confidence interval (2 s.e.m.) were calculated. Differences in response between groups and controls were evaluated by ANOVA.

### RESULTS

### Alterations in vessel diameter

Acute changes in the diameter of 20–30 μm arterioles and venules in cremaster muscle and changes in 20–30 μm tumour microvasculature were monitored through PDT treatment and for a period of 60 min after the completion of treatment. Administration of light doses up to 150 J $cm^{-2}$ 690 nm caused a slight dilation of arterioles early after the initiation of PDT, but this was not statistically different than controls (Figure 1A). No change in the diameter of cremaster venules was observed irrespective of the time between BPD injection and light treatment (Figure 1B). No statistically

*© Cancer Research Campaign 1999*

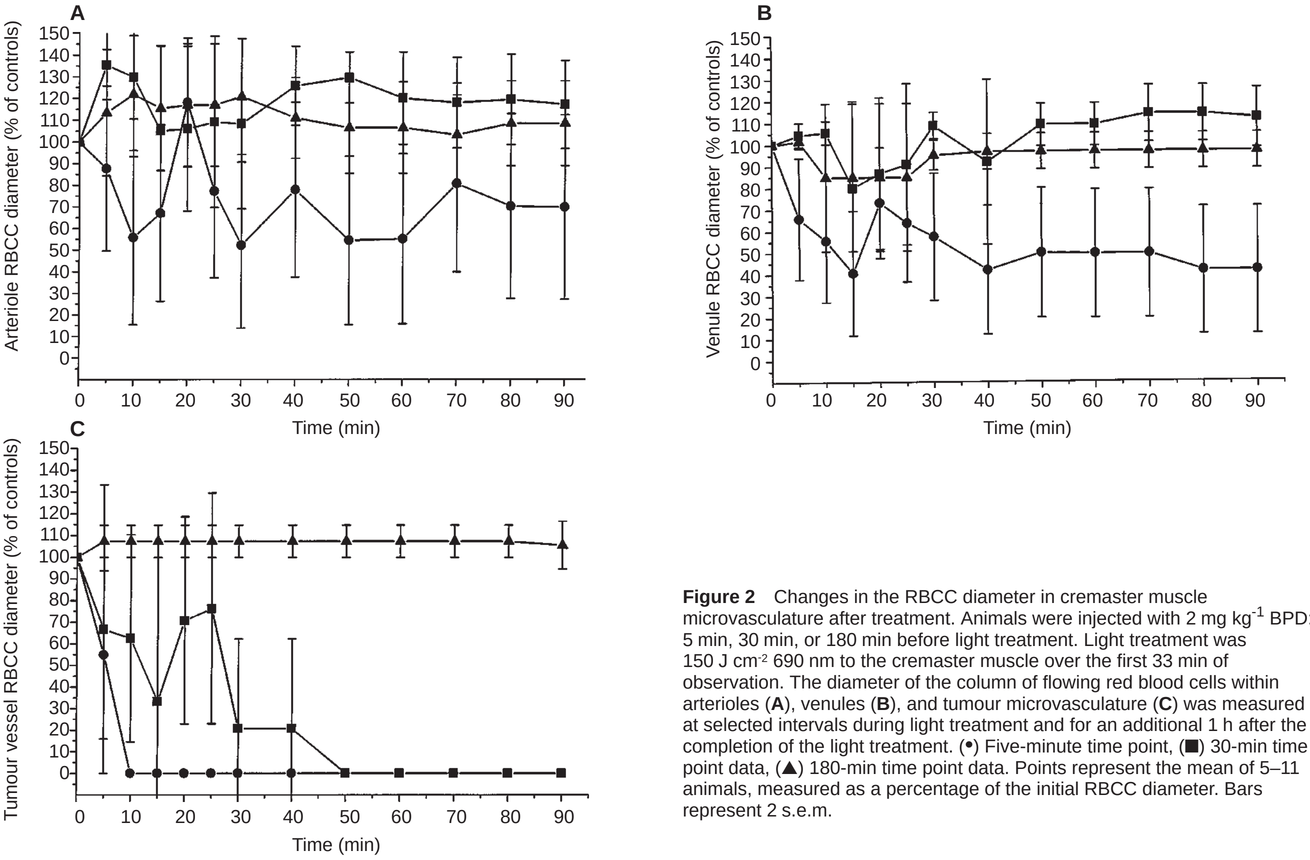

**Figure 2** Changes in the RBCC diameter in cremaster muscle microvasculature after treatment. Animals were injected with 2 mg $kg^{-1}$ BPD: 5 min, 30 min, or 180 min before light treatment. Light treatment was 150 J $cm^{-2}$ 690 nm to the cremaster muscle over the first 33 min of observation. The diameter of the column of flowing red blood cells within arterioles (**A**), venules (**B**), and tumour microvasculature (**C**) was measured at selected intervals during light treatment and for an additional 1 h after the completion of the light treatment. (•) Five-minute time point, (■) 30-min time point data, (▲) 180-min time point data. Points represent the mean of 5–11 animals, measured as a percentage of the initial RBCC diameter. Bars represent 2 s.e.m.

significant change in tumour microvascular diameter was observed in animals given treatment at 5 min, 30 min, or 180 min after BPD injection (Figure 1C).

### Alterations in the luminal diameter of microvasculature

The diameter of the flowing column of red blood cells was monitored in the same microvasculature described in the previous section and represents the effective luminal diameter of the blood vessels. Significant reductions of up to 50–60% were observed in animals given PDT 5 min after BPD injection (Figure 2A,B) ($P < 0.05$ for arterioles, $P < 0.01$ for venules). Blood flow stasis did not occur. Animals treated after longer BPD incubation times did not exhibit this response in the normal microvasculature. Complete stasis of blood flow and loss of the vessel lumen was observed in tumour from animals given light treatment within 30 min of BPD injection (Figure 2C). Vascular stasis occurred within 45 J $cm^{-2}$ light exposure in animals given treatment at 5 min after BPD injection. In PDT protocols using 5 and 30 min BPD incubation times, no reperfusion in the tumour microvasculature was observed within 1 h after the completion of light treatment compared to animals in the 180-min protocol group or controls ($P < 0.05$). Blood flow stasis was absent in tumour microvasculature when tissues were illuminated 180 min after BPD injection.

### Vessel histology

Damage to normal microvasculature in cremaster muscle was evaluated histologically to determine the cause of reductions in vessel lumen diameter in the intravital microscopy experiments (Figure 3A–D). Extensive platelet aggregation and red blood cell agglutination was observed in tissue samples from animals given light treatment 5 min after BPD injection (Figure 3B). This response was diminished in the 30-min treatment group although foci of platelet aggregates at the vessel wall could still be observed (Figure 3C). No apparent damage was observed in the 180-min incubation group compared to controls (Figure 3D).

### Fluorescein exclusion

A qualitative assessment of vessel shutdown in subcutaneously implanted tumour and surrounding tissue was performed using a fluorescein dye exclusion study. Exclusion of this fluorescent dye in vessels indicates that vascular stasis has occurred, reductions in the intensity of dye in tissues correlates with reduced tumour perfusion. When the animals were injected with fluorescein immediately after light treatment and observed under the ultraviolet light, no fluorescence was observed in tumour from animals given BPD either 5 min or 30 min before treatment. Fluorescein was not excluded in the tumour or the untreated leg or in control animals. Treatment of animals 180 min after BPD injection did not result in any exclusion of the fluorescein dye in the tumour area immediately after PDT which correlates with the cremaster studies. When assayed 24 h after the completion of PDT treatment, complete dye exclusion was still observed in the 5 min BPD incubation group; however, visible reductions in the intensity of fluorescein fluorescence were also observed in animals in the 180-min treatment group indicating that some degree of late vascular damage and/or

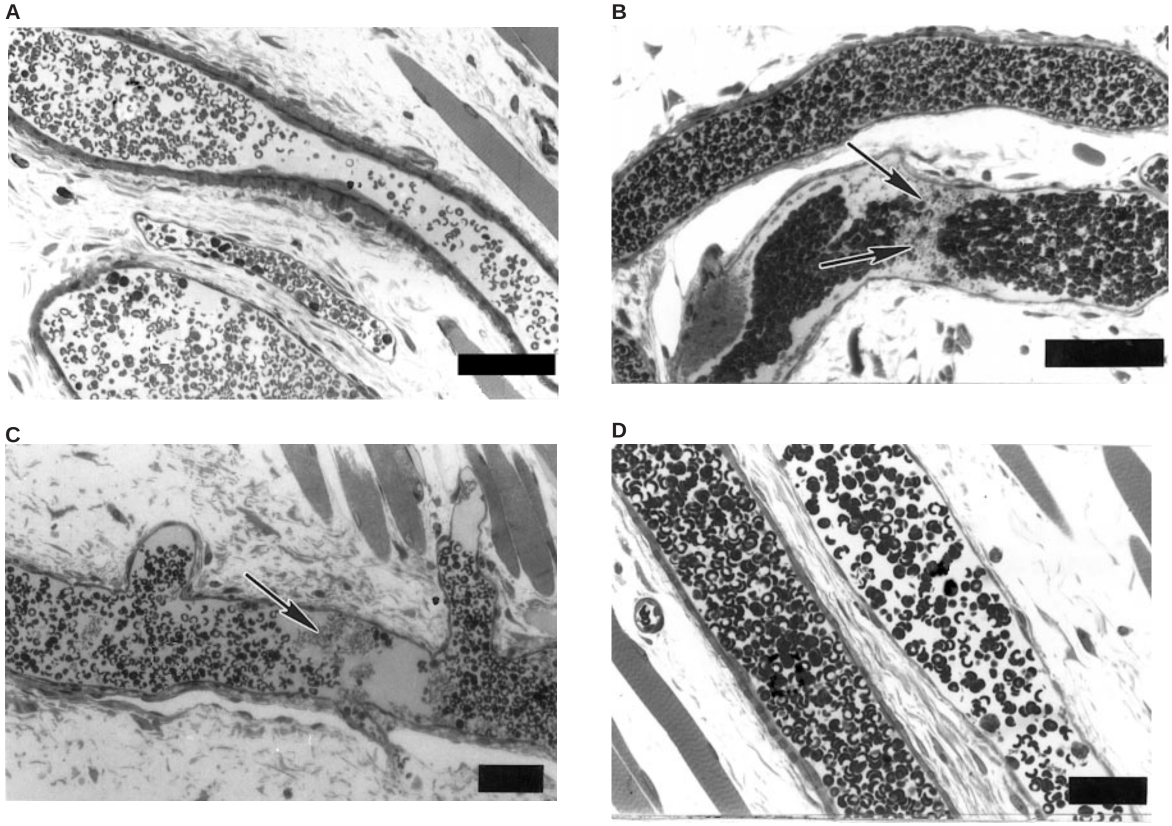

**Figure 3** Histological studies. Animals were injected with 2 mg $kg^{-1}$ BPD i.v. and given light treatment after either 5 min, 30 min or 180 min. Light treatment was 150 J $cm^{-2}$ 690 nm to the cremaster muscle. Tissue samples containing pairs of arteriole and venules were collected 1 h after the completion of the light treatment. (**A**) Sham control, 675 × magnification. (**B**) Animals given light treatment 5 min after BPD injection (800 × magnification). (**C**) Animals given light treatment 30 min after BPD injection (475 × magnification). (**D**) Animals given light treatment 180 min after BPD injection (530 × magnification). In Figures, arrows indicate the location of platelet aggregation, bar represents 20 μm blood flow stasis in these animals occurs. In order to further evaluate the effect in this set of animals, the fluorescence signal from tumour was digitized and gray scale analysis was performed. Animals given BPD and light 180 min after injection had gray scale levels for tumour of 107.1 ± 22.4 (mean ± 2 s.e.m.) when assayed 24 h after PDT. This value is statistically less than that for control tumours given BPD and no light (190.4 ± 16.2, $P = 0.0002$), indicating a reduction in perfusion in tumour for the PDT-treated animals at 24 h. The progression of vascular damage in these treated animals did not result in complete blood flow stasis since even lower gray levels could be produced in tumour from animals given light treatment 5 min after BPD injection (74.9 ± 15.8, $P = 0.035$) where stasis was confirmed by intravital microscopy. Background gray levels of tumour from animals given no fluorescein were 60.7 ± 4.3.

### Tumour response

The efficacy of PDT using BPD for the different treatment times of 5 min, 30 min and 180 min after photosensitizer injection was evaluated using a subcutaneously implanted chondrosarcoma model (Figure 4). Treatment produced initial regression of tumour in animals given BPD at each of the dosing protocols, tumours were flat and apparently necrotic within 48 h of light treatment. The durability of long-term control of tumour regrowth was inversely proportional to the time between BPD injection and light treatment. Regrowth of tumour in certain animals in the 30 min and 180 min BPD incubation groups was observed after 15 days. Five percent of the animals treated with 2 mg $kg^{-1}$ BPD 180 min prior to 150 J $cm^{-2}$ were tumour-free after 42 days. Fifty-five percent of the animals treated with 2 mg $kg^{-1}$ BPD 30 min before 150 J $cm^{-2}$ light dose were free of tumour after 42 days. All animals given light treatment 5 min after BPD injection remained tumour-free at 42 days post-treatment. There was significant normal tissue damage and necrosis in the 5-min group, with heavy oedema and eschar forming 1-day post-treatment. Damage was extensive in these animals resulting in irreversible damage to muscle in the treatment field. Tumour destruction was more selective in animals given light treatment at longer BPD incubation periods. No eschar or apparent damage to skin or muscle was observed in animals in the 180-min protocol group. Normal tissue damage in the 30-min group did occur, but this was not as extensive as in the 5-min group.

### DISCUSSION

The relative effect of PDT using BPD-MA in different dosing regimens on damage to tumour and normal microvasculature was evaluated and correlated with tumour regression.

© Cancer Research Campaign 1999

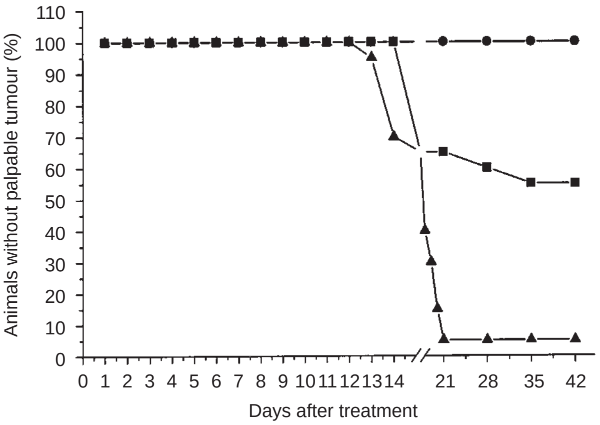

**Figure 4** Tumour response and cure following PDT with BPD. Sprague–Dawley rats were injected with 2 mg $kg^{-1}$ BPD i.v. at 5 min, 30 min or 180 min before light treatment. Light treatment was 150 J $cm^{-2}$ 690 nm to a 1.5 cm diameter spot centred over chondrosarcoma on the right hind limb. Animals were considered cured of tumour if no regrowth was observed before 42 days after treatment. (•) Animals given light treatment 5 min after BPD injection (*n* = 5), (■) animals given light treatment 30 min after BPD injection (*n* = 20), (▲) animals given light treatment 180 min after BPD injection (*n* = 20)

An acute vascular reaction to PDT was found in microvasculature during intravital microscopy and the magnitude of this event was greatest when light treatment was delivered at short times after BPD injection. At short, 5 min, incubation times, this response was characterized by reductions in the luminal diameter of microvasculature due to the formation of platelet aggregates at the vessel wall. Aggregate formation in tumour microvasculature continued through light treatment until the vascular lumen was completely occluded. Constriction of the wall of blood vessels was not observed during, or acutely after, PDT as found with certain photosensitizers including Photofrin (Fingar et al, 1992). Similar effects were found in tumour microvasculature for animals given light treatment after 30 min BPD incubation. Complete or nearly complete occlusion of microvasculature in tumour was observed by the end of the light treatment; however, no pronounced changes were observed in normal blood vessels in muscle adjacent to the tumour indicating that selectivity for damage could be obtained at the 30-min time point. Light treatment 180 min after BPD administration produced no apparent acute vascular response in either normal muscle microvasculature or tumour blood vessels. Although light treatment did not produce acute microvascular damage and blood flow stasis in tumour, damage to these blood vessels did progress over the next 24 h and did result in significant reductions in tissue perfusion as shown by the fluorescein dye exclusion studies. Progressive damage to tumour microvasculature after the completion of PDT using BPD has been described by Levy et al (1993).

These data were confirmed by histology of tissue sections taken immediately after PDT. Foci of platelet aggregates were observed in arterioles and venules in animals given light treatment at 5 or 30 min after BPD injection. Likewise, analysis of fluorescein dye exclusion revealed complete blood flow stasis in treated microvasculature immediately after PDT for animals given light 5 min after BPD injection, but not at the 180-min BPD incubation interval.

The formation of platelet thrombi leading to vessel occlusion and blood flow stasis after PDT using BPD that was observed in these studies of tumour microvasculature has been described in several studies of rabbit eye microvasculature and ocular tumours. Lin et al (1994) observed the formation of platelet debris and extravasation of red blood cells in choroidal capillaries after treatment with light within 1 min of BPD injection. The vascular endothelium was significantly damaged with loss of cellular structure and the collagen matrix surrounding vessels was disorganized. Selective thrombosis in corneal vasculature and subretinal vessels was observed after fluorescein angiography in studies by Schmidt-Erfurth et al (1994, 1995) using BPD at 180 min. Vascular damage and ischaemia resulting from endothelial cell damage and platelet aggregation has also been described in intraocular tumours after PDT using BPD with incubation times of 15 and 180 min (Schmidt-Erfurth et al, 1996; Young et al, 1996).

The time course of the vascular photosensitization in the rat cremaster muscle with chondrosarcoma differed from the data from the rabbit eye models described by Schmidt-Erfurth et al (1994, 1995) since we did not observe acute vascular damage in either normal muscle tissue or in tumour at 180 min. The reasons for this difference remain unclear but may be related to species differences in plasma pharmacokinetics or tissue-specific differences in BPD retention. Overall, the data from our studies and the previous work in the rabbit models demonstrate that vascular damage after PDT is greater in animals given light treatment at short intervals after BPD injection. These data are consistent with measurements of tissue levels of BPD in highly vascularized structures of the rabbit eye such as the cilliary body and choroid where the highest BPD levels were observed 5 min after photosensitizer injection (Haimovici et al, 1997) and the rapid distribution of BPD into murine tissues after injection (Richter et al, 1990, 1993).

The selectivity in tumour microvascular response that we observed after the 30-min BPD incubation period compared to earlier treatment points is similar to the selectivity observed in rabbit ocular tissues at 180 min BPD incubation (Schmidt-Erfurth et al, 1994, 1995). This selectivity may be related to the increased accumulation of BPD by tumour endothelial cells with high levels of low-density lipoprotein (LDL) receptors (Allison et al, 1994) or the rapid internalization of agents bound to the LDL receptor on proliferating endothelial cells compared to quiescent endothelial cells (Fielding et al, 1979). Cells with increased phagocytotic activity, such as injured vessel wall in atherosclerotic vessels, have been shown to accumulate BPD at levels in excess of surrounding microvasculature (Allison et al, 1997) and this may play a role in tumour microvasculature as well. Similarly, macrophages show pronounced levels of BPD uptake compared to other cells and this may also contribute to the observed effect (Korbelik and Krosl, 1995).

Tumour regression and the degree of surrounding tissue damage after PDT correlated with the presence or absence of acute vascular stasis observed in the rat cremaster model. Tissues that exhibited early vascular stasis and ischaemia showed significant damage 24–48 h after PDT with tissue destruction and eschar formation. Although initial regression occurred in chondrosarcoma treated with BPD at all three incubation protocols, including the 180-min incubation protocol where no initial vascular stasis was observed during intravital microscopy, the greatest number of animals that remained tumour-free at 42 days was with the 5-min incubation protocol. This effect may be related to the absence of tissue selectivity in this treatment protocol and that a margin of normal tissue must be destroyed to ensure tumour cure (Fingar and Henderson, 1987; Star et al, 1986). The necrosis in the skin surrounding tumour and the underlying muscle in these animals is apparently due to tissue ischaemia as predicted by the microvascular studies.

*© Cancer Research Campaign 1999* 

Significant delays before tumour regrowth were observed in the animals given light treatment 180 min after BPD injection and this may be related to the observations of late vascular stasis in animals treated with this protocol. The timing of tumour regrowth in these animals is consistent with the presence of surviving nests of tumour cells after PDT that exist close to the undamaged surrounding normal tissues and cause regrowth at a later time.

An important conclusion of these studies is that the timing of light treatment after BPD injection is critical and that the magnitude of vascular damage in different tissues can be modulated by treating at earlier or later times. For tumour destruction, treatment times can be chosen to balance the relative selectivity of the tissue ablation. Vessel damage and blood flow stasis is a significant component of the PDT effect that contributes to tumour regression, and early treatment times can be chosen to maximize this effect. These data may have important implications for treatment of non-cancer applications in which vessel ablation is desired. For treatment of aberrant neovasculature in tissues, including the eye where selectivity is vital, treatment should be delivered at later periods after photosensitizer injection where selectivity is observed rather than treating immediately after BPD administration. Since the relative clearance and metabolism of BPD varies in different tissues and in different species, additional investigations are warranted to identify the timing for optimal vascular sensitivity in humans.

### ACKNOWLEDGEMENTS

This investigation was supported by PHS Grant Number CA51771 and CA65561 awarded by the National Cancer Institute, DHHS, by the Department of Surgery and the Henry Vogt Cancer Institute at the James Graham Brown Cancer. The authors wish to thank QLT PhotoTherapeutics Inc. for providing the benzoporphyrin derivative used in this study.

### REFERENCES

Allison BA, Waterfield E, Richter AM and Levy JG (1991) The effects of plasma lipoproteins on in vitro tumor cell killing and in vivo tumor photosensitization with benzoporphyrin derivative. *Photochem Photobiol* **54**: 709–715

Allison BA, Pritchard PH and Levy JG (1994) Evidence for low-density lipoprotein receptor-mediated uptake of benzoporphyrin derivative. *Br J Cancer* **69**: 833–839

Allison BA, Crespo MT, Jain AK, Richter AM, Hsiang YN and Levy JG (1997) Delivery of benzoporphyrin derivative, a photosensitizer, into atherosclerotic plaque of watanabe heritable hyperlipidemic rabbits and balloon-injured New Zealand rabbits. *Photochem Photobiol* **65**: 877–883

Chowdary RK, Ratkay LG, Neyndorff C, Richter AM, Obochi M, Waterfield J and Levy JG (1994) The use of transcutaneous photodynamic therapy in the prevention of adjuvant-enhanced arthritis in MRL/*lpr* mice. *Clin Immunol Immunopathol* **72**: 255–263

Fielding PE, Vlodevsky I, Gospodarowicz D and Fielding CJ (1979) Effect of contact inhibition on the regulation of cholesterol metabolism in cultured vascular endothelial cells. *J Biol Chem* **254**: 749–755

Fingar VH and Henderson BW (1987) Drug and light dose dependence of photodynamic therapy: a study of tumor and normal tissue response. *Photochem Photobiol* **46**: 837–841

Fingar VH, Wieman TJ and Doak KW (1990) The role of thromboxane and prostacyclin release on photodynamic therapy-induced tumor destruction. *Cancer Res* **50**: 2599–2603

Fingar VH, Wieman TJ, Wiehle SA and Cerrito PB (1992) The role of microvascular damage in photodynamic therapy: the effect of treatment on vessel constriction, permeability and leukocyte adhesion. *Cancer Res* **52**: 4914–4921

Haimovici R, Kramer M, Miller JW, Hasan T, Flotte TJ, Schomacker KT and Gragoudas ES (1997) Localization of lipoprotein-delivered benzoporphyrin derivative in the rabbit eye. *Current Eye Res* **16**: 83–90

Henderson BW and Dougherty TJ (1992) How does photodynamic therapy work? *Photochem Photobiol* **55**: 145–157

Henderson BW and Farrell G (1989) Possible implications of vascular damage for tumor cell inactivation in vivo: comparison of different photosensitizers. In *SPIE Proceedings 1065 Photodynamic Therapy*: *Mechanisms*. Dougherty TJ (ed), pp. 2–10. The International Society for Optical Engineering, Bellingham, Washington, USA

Korbelik M and Krosl G (1995) Accumulation of benzoporphyrin derivative in malignant and host cell populations of the murine RIF tumor. *Cancer Lett* **97**: 249–254

Levy JG (1994) Photosensitizers in photodynamic therapy. *Semin Oncol* **2**: 4–10

Levy JG, Waterfield E, Richter A, Smits C, Lui H, Hruza L, Anderson R and Salvatori V (1993) Photodynamic therapy of malignancies with benzoporphyrin derivative monoacid ring A. Proceedings of Photodynamic Thearpy of Cancer Society of Photo-Optical Instrumentaion Engineers, Bellingham, WA. *SPIE* **2078**: 91–98

Lin SC, Lin CP, Feld JR, Duker JS and Puliafito CA (1994) The photodynamic occlusion of choroidal vessels using benzoporphyrin derivative. *Current Eye Res* **13**: 513–522

North J, Coombs R and Levy JG (1994) Photodynamic inactivation of free and cell-associated HIV-1 using the photosensitizer, benzoporphyrin derivative. *J Acquired Immune Deficiency Synd* **7**: 891–898

Richter AM, Cerruti-Sola S, Sternberg ED, Dolphin D and Levy JG (1990) Biodistribution of tritiated benzoporphyirn derivative (3H-BPD-MA), a new potent photosensitizer, in normal and tumor-bearing mice. *J Photochem Photobiol B*: *Biology* **5**: 231–244

Richter AM, Waterfield E, Jain AK, Allison B, Sternberg ED, Dolphin D and Levy JG (1991) Photosensitising potency of structural analogues of benzoporphyrin derivative (BPD) in a mouse tumour model. *Br J Cancer* **63**: 87–93

Richter AM, Waterfield E, Jain AK, Canaan AJ, Allison BA and Levy JG (1993) Liposomal delivery of a photosensitizer, benzoporphyrin derivative monoacid ring A (BPD), to tumor tissue in a mouse tumor model. *Photochem Photobiol* **57**: 1000–1006

Schmidt-Erfurth U, Hasan T, Gragoudas E, Michaud N, Flotte TJ and Birngruber R (1994) Vascular targeting in photodynamic occlusion of subretinal vessels. *Ophthalmology* **101**: 1953–1961

Schmidt-Erfurth U, Hasan T, Schomacker K, Flotte T and Birngruber R (1995) In vivo uptake of liposomal benzoporphyrin derivative and photothrombosis in experimental corneal neovascularization. *Laser Surg Med* **17**: 178–188

Schmidt-Erfurth U, Flotte TJ, Gragoudas ES, Schomacker K, Birngruber R and Hasan T (1996) Benzoporphyrin-lipoprotein-mediated photodestruction of intraocular tumors. *Exp Eye Res* **62**: 1–10

Star WM, Marijnissen HPA, van den Berg-Blok S, Versteeg JAC, Franken KAP and Reinshol HS (1986) Destruction of rat mammary tumour and normal tissue microcirculation by hematoporphyrin derivative photoradiation observed in vivo in sandwich observation chambers. *Cancer Res* **46**: 2532–3540

Wyss P, Tadir Y, Tromberg BJ, Liaw L, Krasieva T and Berns MW (1994) Benzoporphyrin derivative: a potent photosensitizer for photodynamic destruction of rabbit endometrium. *Obs Gynecol* **84**: 409–414

Young LH, Howard MA, Hu LK, Kim RY and Gragoudas ES (1996) Photodynamic therapy of pigmented choroidal melanomas using a liposomal preparation of benzoporphyrin derivative. *Arch Ophthamol* **114**: 186–192

 *© Cancer Research Campaign 1999*

## References

[PDF_00685] Allison B. A., Crespo M. T., Jain A. K., Richter A. M., Hsiang Y. N., Levy J. G. (1997). Delivery of benzoporphyrin derivative, a photosensitizer, into atherosclerotic plaque of Watanabe heritable hyperlipidemic rabbits and balloon-injured New Zealand rabbits.. Photochem Photobiol.

[PDF_00682] Allison B. A., Pritchard P. H., Levy J. G. (1994). Evidence for low-density lipoprotein receptor-mediated uptake of benzoporphyrin derivative.. Br J Cancer.

[PDF_00679] Allison B. A., Waterfield E., Richter A. M., Levy J. G. (1991). The effects of plasma lipoproteins on in vitro tumor cell killing and in vivo tumor photosensitization with benzoporphyrin derivative.. Photochem Photobiol.

[PDF_00689] Chowdhary R. K., Ratkay L. G., Neyndorff H. C., Richter A., Obochi M., Waterfield J. D., Levy J. G. (1994). The use of transcutaneous photodynamic therapy in the prevention of adjuvant-enhanced arthritis in MRL/lpr mice.. Clin Immunol Immunopathol.

[PDF_00693] Fielding P. E., Vlodavsky I., Gospodarowicz D., Fielding C. J. (1979). Effect of contact inhibition on the regulation of cholesterol metabolism in cultured vascular endothelial cells.. J Biol Chem.

[PDF_00696] Fingar V. H., Henderson B. W. (1987). Drug and light dose dependence of photodynamic therapy: a study of tumor and normal tissue response.. Photochem Photobiol.

[PDF_00699] Fingar V. H., Wieman T. J., Doak K. W. (1990). Role of thromboxane and prostacyclin release on photodynamic therapy-induced tumor destruction.. Cancer Res.

[PDF_00702] Fingar V. H., Wieman T. J., Wiehle S. A., Cerrito P. B. (1992). The role of microvascular damage in photodynamic therapy: the effect of treatment on vessel constriction, permeability, and leukocyte adhesion.. Cancer Res.

[PDF_00705] Haimovici R., Kramer M., Miller J. W., Hasan T., Flotte T. J., Schomacker K. T., Gragoudas E. S. (1997). Localization of lipoprotein-delivered benzoporphyrin derivative in the rabbit eye.. Curr Eye Res.

[PDF_00708] Henderson B. W., Dougherty T. J. (1992). How does photodynamic therapy work?. Photochem Photobiol.

[PDF_00715] Korbelik M., Krosl G. (1995). Accumulation of benzoporphyrin derivative in malignant and host cell populations of the murine RIF tumor.. Cancer Lett.

[PDF_00718] Levy J. G. (1994). Photosensitizers in photodynamic therapy.. Semin Oncol.

[PDF_00724] Lin S. C., Lin C. P., Feld J. R., Duker J. S., Puliafito C. A. (1994). The photodynamic occlusion of choroidal vessels using benzoporphyrin derivative.. Curr Eye Res.

[PDF_00727] North J., Coombs R., Levy J. (1994). Photodynamic inactivation of free and cell-associated HIV-1 using the photosensitizer, benzoporphyrin derivative.. J Acquir Immune Defic Syndr.

[PDF_00730] Richter A. M., Cerruti-Sola S., Sternberg E. D., Dolphin D., Levy J. G. (1990). Biodistribution of tritiated benzoporphyrin derivative (3H-BPD-MA), a new potent photosensitizer, in normal and tumor-bearing mice.. J Photochem Photobiol B.

[PDF_00734] Richter A. M., Waterfield E., Jain A. K., Allison B., Sternberg E. D., Dolphin D., Levy J. G. (1991). Photosensitising potency of structural analogues of benzoporphyrin derivative (BPD) in a mouse tumour model.. Br J Cancer.

[PDF_00737] Richter A. M., Waterfield E., Jain A. K., Canaan A. J., Allison B. A., Levy J. G. (1993). Liposomal delivery of a photosensitizer, benzoporphyrin derivative monoacid ring A (BPD), to tumor tissue in a mouse tumor model.. Photochem Photobiol.

[PDF_00747] Schmidt-Erfurth U., Flotte T. J., Gragoudas E. S., Schomacker K., Birngruber R., Hasan T. (1996). Benzoporphyrin-lipoprotein-mediated photodestruction of intraocular tumors.. Exp Eye Res.

[PDF_00741] Schmidt-Erfurth U., Hasan T., Gragoudas E., Michaud N., Flotte T. J., Birngruber R. (1994). Vascular targeting in photodynamic occlusion of subretinal vessels.. Ophthalmology.

[PDF_00744] Schmidt-Erfurth U., Hasan T., Schomacker K., Flotte T., Birngruber R. (1995). In vivo uptake of liposomal benzoporphyrin derivative and photothrombosis in experimental corneal neovascularization.. Lasers Surg Med.

[PDF_00750] Star W. M., Marijnissen H. P., van den Berg-Blok A. E., Versteeg J. A., Franken K. A., Reinhold H. S. (1986). Destruction of rat mammary tumor and normal tissue microcirculation by hematoporphyrin derivative photoradiation observed in vivo in sandwich observation chambers.. Cancer Res.

[PDF_00754] Wyss P., Tadir Y., Tromberg B. J., Liaw L., Krasieva T., Berns M. W. (1994). Benzoporphyrin derivative: a potent photosensitizer for photodynamic destruction of rabbit endometrium.. Obstet Gynecol.

[PDF_00757] Young L. H., Howard M. A., Hu L. K., Kim R. Y., Gragoudas E. S. (1996). Photodynamic therapy of pigmented choroidal melanomas using a liposomal preparation of benzoporphyrin derivative.. Arch Ophthalmol.

